# ‘*Candidatus* Megaira polyxenophila’ gen. nov., sp. nov.: Considerations on Evolutionary History, Host Range and Shift of Early Divergent Rickettsiae

**DOI:** 10.1371/journal.pone.0072581

**Published:** 2013-08-20

**Authors:** Martina Schrallhammer, Filippo Ferrantini, Claudia Vannini, Stefano Galati, Michael Schweikert, Hans-Dieter Görtz, Franco Verni, Giulio Petroni

**Affiliations:** 1 Dipartimento di Biologia, Università di Pisa, Pisa, Italy; 2 Biologisches Institut, Universität Stuttgart, Stuttgart, Germany; 3 Institut für Hydrobiologie, Technische Universität Dresden, Dresden, Germany; University of Melbourne, Australia

## Abstract

“Neglected *Rickettsiaceae*” (*i.e.* those harboured by non-hematophagous eukaryotic hosts) display greater phylogenetic variability and more widespread dispersal than pathogenic ones; yet, the knowledge about their actual host range and host shift mechanism is scarce. The present work reports the characterization following the full-cycle rRNA approach (SSU rRNA sequence, specific *in situ* hybridization, and ultrastructure) of a novel rickettsial bacterium, herewith proposed as '*Candidatus* Megaira polyxenophila' gen. nov., sp. nov. We found it in association with four different free-living ciliates (*Diophrys oligothrix, Euplotes octocarinatus, Paramecium caudatum*, and *Spirostomum* sp., all belonging to Alveolata, Ciliophora); furthermore it was recently observed as intracellular occurring in *Carteria cerasiformis* and *Pleodorina japonica* (Chlorophyceae, Chlorophyta). Phylogenetic analyses demonstrated the belonging of the candidate new genus to the family *Rickettsiaceae* (*Alphaproteobacteria*, *Rickettsiales*) as a sister group of the genus *Rickettsia*. *In situ* observations revealed the ability of the candidate new species to colonize either nuclear or cytoplasmic compartments, depending on the host organism. The presence of the same bacterial species within different, evolutionary distant, hosts indicates that '*Candidatus* Megaira polyxenophila' recently underwent several distinct host shifts, thus suggesting the existence of horizontal transmission pathways. We consider these findings as indicative of an unexpected spread of rickettsial infections in aquatic communities, possibly by means of trophic interactions, and hence propose a new interpretation of the origin and phylogenetic diversification of rickettsial bacteria.

## Introduction

**Figure 1:**
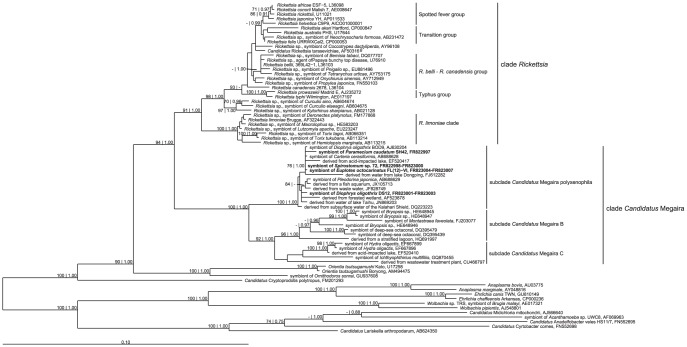
Maximum likelihood phylogenetic tree. Numbers indicate bootstrap values inferred after 1,000 pseudoreplicates and Bayesian Posterior Probabilities (values below 70% respectively 0.7 are not shown). Sequences characterized in the present work are reported in bold. Scale bar: 10 nucleotide substitutions per 100 positions.

The family *Rickettsiaceae* (*Alphaproteobacteria*, *Rickettsiales*, [1]) comprises 28 validly described species, subdivided into the genera *Rickettsia* and *Orientia* (reviewed by [2]). Furthermore, it contains an increasing number of recently discovered organisms, either belonging to the same or to different genera, which are still lacking a formal description or which have been proposed as candidate novel species (e.g. [3]–[8]). Additionally, an increasing amount of associated sequences derives from screenings of environmental microbial populations (Fig. 1, [9], [10]). As far as all species comprised within the family *Rickettsiaceae* share an obligate intracellular lifestyle, we conjecture that the bacteria represented by those sequences are indeed not free-living organisms occurring in the screened habitats but unrecognized endosymbionts.

Many *Rickettsiaceae* are noticed for causing human diseases. Pathogenic *Rickettsiaceae*, e.g. the causative agents of Rocky Mountain spotted fever (*Rickettsia rickettsii*), epidemic typhus (*Rickettsia prowazekii*), murine typhus (*Rickettsia typhi*), and scrub typhus (*Orientia tsutsugamushi*) have been extensively studied because of their medical relevance. On the other hand, little is known about the other species. Present knowledge about this family is mainly congruent with that concerning pathogenic *Rickettsia* and *Orientia*. Yet, the “neglected *Rickettsiaceae*”, *i.e. Rickettsia*-like bacteria of less clinical concern, associated with non-hematophagous arthropods and/or with other eukaryotes, should be subject of considerable interest. First, this group accounts for the main contribution to the evolutionary diversity of *Rickettsiaceae*, gathering 10 out of the 13 phylogenetic clusters comprised within that family (Fig. 1; [9]). Second, these bacteria display a broad host range, including arthropods (reviewed by [11]), protists [12]–[19], cnidarians [20], leeches [21] and even plants [22], [23], while medically relevant rickettsiae are typically confined to ticks or lice [2] with vertebrates as alternate hosts. Third, some “neglected *Rickettsiaceae*”, e.g. those associated with *Acyrthosiphon pisum*, *Empoasca papayae*, or *Bemisia tabaci*, have been recognized as pathogens or parasites of their eukaryotic hosts [22], [24], [25]. A continued and extended characterization also of these groups of *Rickettsiaceae* will increasingly strengthen rickettsial phylogenetic reconstructions, providing a more realistic model of the evolutionary history of the group. This will allow at the same time to elucidate the evolutionary pattern of some typical rickettsial traits, such as the monoxenic or polyxenic life cycle and the development of pathogenic capabilities.

In particular, an improved knowledge of “neglected *Rickettsiaceae*” will allow a better understanding how rickettsiae developed their transmission mechanisms between different hosts. These bacteria seem to rely on different transmission strategies than pathogenic *Rickettsia* and *Orientia* which are both known to exploit blood-sucking arthropods for ensuring vertical and horizontal transmission [26]. “Neglected *Rickettsiaceae*” are harboured by non-hematophagous hosts, thus are generally considered to be strictly vertically transmitted [27]. This idea was supported by the fact that such rickettsial species were never found infecting two or more different hosts.

Improvements in sampling, however, are drawing a different situation. Weinert and colleagues [9] demonstrated that numerous host shifts occurred within phylogenetically related rickettsial species during their evolution; moreover, closely related rickettsiae do not always share similar hosts, but *Rickettsiaceae* living in the same habitat are frequently found infecting unrelated hosts which share similar ecological niches. Thus, it appears that rickettsial diversification mainly relies on infection of different hosts inhabiting the same natural environment rather than on co-speciation processes originating from an ancient symbiotic relationship [9]. This model implicates that some “neglected *Rickettsiaceae*” species would potentially still retain the capability to infect different eukaryotic hosts, either permanently or in a transitional way.

The present work describes intracellular bacteria found in different ciliate hosts belonging to four distinct and geographically distant populations. Phylogenetic reconstructions demonstrated their close relationship with intracellular bacteria of two members of Chlorophyta [10] and another, formally not yet described ciliate endosymbiont. Therefore, the latter [28] has been included in the present work in order to update the analysis, to confirm its ultrastructural morphology and to provide a formal description of this novel species. The improved phylogenetic reconstructions show the seven sequences clustering as different strains of a novel *Rickettsiaceae* species, herewith proposed as '*Candidatus* Megaira polyxenophila'. Fluorescence *in situ* hybridizations and ultrastructural observations are included in the description of these organisms. The possible implications of the finding of these strains harboured by several different hosts for evolutionary history, host range and host shift mechanisms of *Rickettsiaceae* are addressed.

## Material and Methods

### Host isolation, cultivation, and identification

All ciliate strains and populations were collected from public areas with no restrictions of access (e.g. bathing areas). Genus- or species-level classification of the ciliate hosts harbouring the here characterized rickettsial bacteria was achieved by exploiting morphological features as taxonomic markers, and then confirmed by SSU rRNA gene sequence analysis.

Total DNA was extracted from ca. 50 specimens for each strain. Before fixation, cells were repeatedly rinsed in sterile water to minimize bacterial contaminants and salt concentration. Cells were then fixed in 70% EtOH and stored at −20 °C. Before use, ethanol was decanted and samples were dried. Total DNA extraction was achieved through NucleoSpin™ Plant DNA Extraction Kit (Macherey-Nagel GmbH & Co., Düren NRW, Germany), following the CTAB protocol for mycelium. SSU rRNA genes of host ciliates were amplified using the primer pair 18S F9 Euk (*5* ′-CTGGTTGATCCTGCCAG-*3*′; [29]) and 18S R1513 (*5*′-TGATCCTTCYGCAGGTTC-*3*′; [30]). PCR reactions were performed with the annealing taking place at 57 °C for 35 cycles. Resulting PCR products were directly sequenced with appropriate internal primers [31].

#### Diophrys oligothrix BOD9

The strain BOD9 was isolated in 1999 from a brackish environment in Boderne (Bornholm, Baltic Sea). It was identified and cultivated as described elsewhere [28] where it is erroneously reported as *Diophrys appendiculata*.

#### Diophrys oligothrix DS12/4

Ciliate specimens were sampled from a coastal brackish pond termed “Stagno 2” (43°47′16′′ N – 10°16′02′′ E) near Serchio River estuary (Tuscany, Italy). Cells were selected on basis of gross morphological features and isolated in sterile water supplemented with Red Sea Salt™ (Foster and Smith, Rhinelander, US-WI) to reach 5‰ salinity. Cells were regularly fed with *Dunaliella salina* raised at the same salinity conditions [32]. Ten monoclonal strains were obtained. Data presented here refer to the monoclonal strain DS12/4.

#### Euplotes octocarinatus

*E. octocarinatus* FL(12)-VI (originally sampled from Burnell, Florida, USA) has been kindly provided by F. Dini and G. Di Giuseppe (Università di Pisa). Ciliates were cultured at 20 °C in a synthetic medium [33] with the autotrophic flagellate *Chlorogonium elongatum* as food source [34]. The characterization of this strain has been previously published [35].

#### Paramecium caudatum

The monoclonal strain SH42 was obtained from a water sample collected at the Langsee at Suederfahrenstedt (Schleswig-Holstein, Germany). The cells were cultured in Sonneborn's *Paramecium* medium (ATCC medium 802: Cerophyll 2.5 g, Na_2_HPO_4_ 0.5 g, dist. water 1 l) inoculated with food bacteria (*Raoultella planticola*) and maintained at a constant temperature of 20°C.

#### Spirostomum sp

A dense population of *Spirostomum* sp. was observed in a water and sediment sample derived from the Plusssee (Ploen, Schleswig-Holstein, Germany). All attempts to establish a monoclonal culture of these cells failed. *Spirostomum* cells survived in different media (with or without addition of food bacteria) for a maximum period of 14 days. Nevertheless, the ciliate population termed “72” remained stable for ca. three months in the original sample. For experiments, 5–50 cells were isolated and washed several times in sterile spring water (Volvic® mineral, Danone Waters, Frankfurt, Hesse, Germany).

### Molecular characterization of symbionts

The molecular characterization of the bacteria associated with *D. oligothrix* BOD9 was achieved elsewhere [28]. In the other four ciliate strains, the presence of rickettsial symbionts was assessed by *in situ* hybridizations with the universal bacterial probe EUB338 [36] in combination with a probe detecting rickettsial bacteria (probe Rick_527, specific for members of the *Rickettsiaceae* family and *Caedibacter caryophilus*; [28]). Fluorescence *in situ* hybridization (FISH) experiments were carried out according to Manz and colleagues [37], without formamide in the hybridization buffer.

SSU rRNA bacterial genes were amplified using the primers 16 S Alfa F19a (*5′* -CCTGGCTCAGAACGAACG-*3′* ) and 16 S Alfa R1517 (*5′* -TGATCCAGCCGCAGGTTC-*3′* ), both targeting the *Alphaproteobacteria* group [14]. To increase the specificity of the reaction and minimize the possibility of amplification errors, high-fidelity *Taq* was always used (TaKaRa Ex *Taq*, Takara Bio Inc., Otsu, Japan), and PCR reactions were set as touchdown PCR, with the annealing taking place at 63°C for the first 5 cycles, 57°C for the next 10 cycles, and 50 °C for the last 25 cycles. The purified reaction product was subjected to cloning with the TOPO TA Cloning® kit (Invitrogen, Carlsbad, US-CA) and ligated with the vector pCR2.1-TOPO™. Chemically competent *Escherichia coli* TOP10 cells (Invitrogen) were transformed according to the manufacturer's instructions. Screening of clone libraries was performed through a low-stringency PCR reaction against the inserted fragment, using vector specific M13 forward and M13 reverse primers (Invitrogen), followed by the digestion of obtained amplicons with *Rsa*I restriction enzyme (Fermentas, Burlington, Canada) for *D. oligothrix* DS12/4, with *Bsp143*I (Fermentas) for *Spirostomum* sp. 72, and with *Bsu*RI (Fermentas) for *E. octocarinatus* FL(12)-VI. Restriction fragment electrophoresis revealed the presence of two main restriction patterns (69.2% and 30.7%, respectively) in the case of *D. oligothrix* DS12/4 and *Spirostomum* sp. 72 (47.4% and 39.5%, respectively), and of one prevalent pattern (53.3%) in the case of *E. octocarinatus* FL(12)-VI. Plasmid DNA was extracted (Quantum Prep™ Plasmid Miniprep kit; BioRad, Hercules, US-CA) and sequenced (Eurofins MWG Operon) from clones showing the dominant restriction patterns. Obtained sequences were compared with each other in order to obtain a consensus. A different strategy was pursued in the case of the rickettsial symbiont of *P. caudatum* SH42. Its SSU rRNA gene was amplified with the primer 16 S F35 OdyHolo (*5* ′*-*GCTGGCGGCATGCTTAAC*-3*′) and 16 S R1488 Holo (*5* ′-TACCTTGTTACGACTTAACC-*3*′; [38]) and then subjected to semi-nested PCRs with primers 16 S F114 HoloCaedi (*5* ′-TGAGTAACGCGTGGGAATC-*3*′; [38]) and 16 S R1328 HoloCaedi (*5* ′-TAGCGATTCCAACTTCATG-*3*′; [38]) to obtain sufficient DNA amounts for direct sequencing. The internal primers were also used for sequencing.

A final FISH experiment was carried out with the specifically designed probe MegPol436 (*5* ′-TTATCTTTCCAACTAAAAG-*3*′) in order to assess the belonging of the characterized sequences to the bacterium of interest. For the determination of additionally present bacterial symbionts, further FISH experiments were performed with probe MegPol436 used in parallel with the following probes: EUB338 [36], Poly_862, specific for the genus *Polynucleobacter* [39], CC23a [40] specific for *Caedibacter caryophilus*, and HoloCar698 (*5* ′-TATTCCTCCTAATATCTGCGA-*3*′) specific for *Holospora caryophila*. The FISH hybridizations were carried out in a buffer containing no formamide according to the recommendations for probes Poly_862, CC23a, MegPol436, and Rick_527. Probe EUB338 is generally used with formamide concentrations ranging from 0-50%; in this study 0% was applied.

### Phylogenetic reconstructions

Phylogenetic analysis was performed by using both the data obtained as described above, and the sequence of the previously characterized rickettsial endosymbiont (AJ630204) of *D*. *oligothrix* BOD9. Sequences were aligned using a sequence editor and aligner (ARB program package [41]), then inserted in a SSU database (SILVA version 111, release July 2012, [42]). The automated alignment was corrected by hand to optimize the base-pairing scheme of the rRNA secondary structure [43]. A filter was constructed on the used selection of sequences to specifically trim upstream and downstream uncharacterized regions. The resulting alignment contained 1,352 columns that were used for phylogenetic analyses. Accession numbers of SSU sequences used in the present work are reported in Fig. 1. Maximum likelihood phylogenetic analysis was performed using the PHYML program [44] from the ARB package [41]; Bayesian Inference analysis was performed with MrBayes [45], using four different Markov Chain Monte Carlo runs, with one cold chain and three heated chains each, running for 1,000,000 generations. The stability of the Maximum likelihood tree was warranted by bootstrap analysis (1,000 pseudoreplicates). A similarity matrix was calculated [46] comprising all sequences affiliated to the candidate new genus.

### TEM preparations

Specimens of *D*. *oligothrix* BOD9, which is infected solely by the bacterium of interest [28], were treated for transmission electron microscopy (TEM) observations. That strain was chosen in order to avoid both misidentifications between the latter and additionally present intracellular bacteria, and alterations in their shape due to eventual interactions with other symbionts. Cells were fixed in 2.5% glutaraldehyde and 1% OsO_4_ in cacodylate buffer 0.05 M, pH 7.4, then dehydrated by passages in ethanol solutions at increasing percentages, and finally embedded in Epon812 resin (Electron Microscopy Science, Hatfield, US-PA). Thin sections were stained as described elsewhere [28], and observed with a JEOL 100 S (Jeol Ltd., Tokyo, Japan).

## Results

### Host identification

Obtained eukaryotic SSU sequences have been deposited to the European Nucleotide Archive (accession numbers: HE664171, *P. caudatum* SH42; HE664172, *D. oligothrix* DS12/4; HE664173, *Spirostomum* sp. 72; HE664174, *D. oligothrix* BOD9). Both morphological observations and SSU rRNA similarity congruently identified the strains SH42 as *P. caudatum*, and FL(12)-VI as *E. octocarinatus*. Gross morphology of strains DS12/4 and BOD9 correspond to the species *D. oligothrix* [47]. 18 S rRNA gene data show 5 bp difference between the two strains and a similarity higher than 99% with other *D. oligothrix* sequences [47], [48]. The morphology of this *Spirostomum* population 72 shared features with *Spirostomum teres* and, to a greater extent, with *Spirostomum minus*, but an unambiguous identification was not achieved. Molecular data placed this population closer to *S. minus* than to other species of the genus, but the differences in the SSU rRNA gene sequence did not support a clear identification of population 72 as *S. minus* (data not shown).

All the ciliate strains and populations used in the study maintained the association with the bacterium of interest in laboratory conditions, except strain DS12/4, which lost it after one month of cultivation. After that period, the absence of bacteria was confirmed by FISH (data not shown).

### Endosymbiont identification

The intracellular bacteria were characterized following the full cycle rRNA approach modified from [49]. SSU rRNA gene sequences of the *Rickettsiaceae* related bacteria were obtained by selective amplification of the target gene and direct sequencing in case of strain SH42, or by cloning and sequencing of clones belonging to the same restriction pattern group (see Material and Methods). In detail, four clones were sequenced for *E. octocarinatus* FL(12)-VI, and three clones for both *Spirostomum* sp. 72 and *D. oligothrix* DS12/4. Differences between clones are reported in Table 1.

**Table:** Table 1. Synopsis of inter-clonal differences. Similarity matrix of bacterial SSU sequences.

Host	Clone	Accession number	Position of varying nucleotide						
*Diophrys oligothrix* DS12/4			**146 (110)**	**304 (257)**	**768 (693)**	**1205 (1131)**	**1329 (1254)**	**1393 (1318)**	**1414 (1339)**
	E1	FR823001	G	G	G	T	A	C	T
	E5	FR823002	A	T	A	T	A	T	C
	F5	FR823003	G	T	A	C	G	T	C
*Spirostomum* sp. 72			**44 (9)**	**91 (55)**	**116 (80)**	**137 (101)**	**672 (597)**	**813 (738)**	
	18	FR822998	G	T	T	T	T	T	
	30	FR822999	A	A	A	C	C	A	
	19	FR823000	A	A	A	T	T	T	
*Euplotes octocarinatus* Fl(12)-VI			**973 (895)**						
	4c	FR823005	C						
	4f	FR823006	C						
	11c	FR823007	C						
	3c	FR823004	/						

Numbers without brackets refer to the position of varying nucleotide with respect to *E. coli* SSU rRNA gene sequence. Numbers in brackets indicate the position of the same nucleotide on the sequence of the clone.

All characterized bacterial SSU gene sequences were submitted to the European Nucleotide Archive and are available with following accession numbers: FR822997, endosymbiont of *P. caudatum* SH42; FR822998 - FR823000, endosymbiont of *Spirostomum* sp. 72, clones 18, 19, 30 respectively; FR823001 - FR823003, endosymbiont of *D. oligothrix* DS12/4, clones E1, E5, F5; FR823004 - FR823007, endosymbiont of *E. octocarinatus* FL(12)-VI, clones 3c, 4c, 4f, 11c.

The probe MegPol436 was specifically designed for the complementation of the full cycle rRNA approach and was *in silico* tested against the SSU rRNA gene sequences (minimum length 1,200 bp) present in the Ribosomal Database project (RDP) [50]. Eleven hits were found, all belonging to *Rickettsiaceae* with one exception. Of the matches identified by RDP, eight correspond to unclassified *Rickettsiaceae* (AJ63020, AF523878, DQ223223, JF828749, JN869203, AB688628, AB688629, JX105713), the other two to members of the genus *Orientia* (EF520417, FJ612282). In phylogenetic reconstructions, all these sequences affiliate with the here-described symbionts in a single subclade (Fig. 1). The single matched sequence outside the *Rickettsiaceae* is classified, according to RDP, as *Burkholderiales incertae sedis* (FJ612303); indeed a closer analysis of this sequence reveals its chimerical origin with the *5*′-end of the sequence (including the target site of probe MegPol436) deriving from an organism belonging to the above mentioned subclade (Fig. 1), and the *3*′-end affiliating with a member of *Betaproteobacteria* (data not shown).

**Figure 2:**
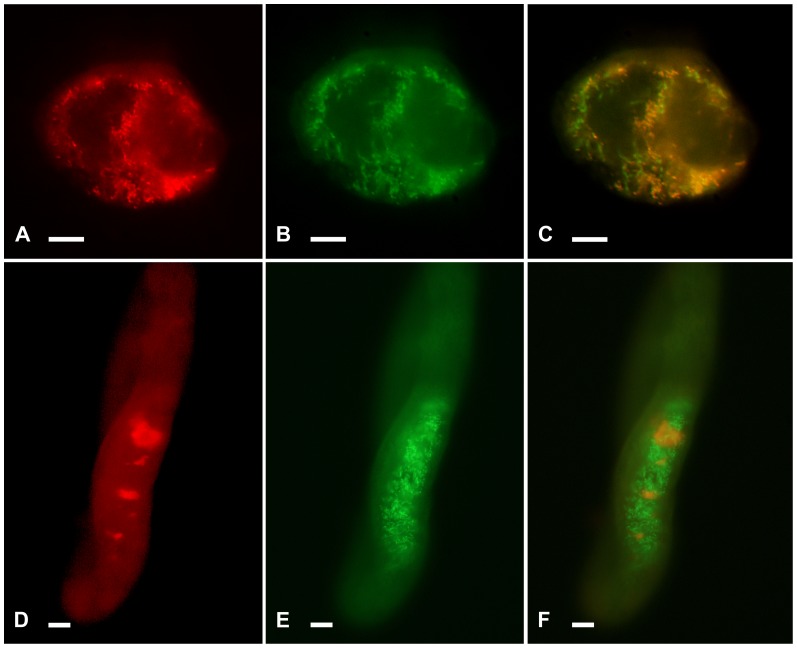
Fluorescence *in situ* hybridizations of *Diophrys oligothrix* DS212/4 and *Paramecium caudatum* SH42. *Diophrys oligothrix* DS212/4 was fixed with 4% formaldehyde in PBS (a-b-c) and *Paramecium caudatum* SH42 was fixed with 4% paraformaldehyde (d-e-f). (a, d) signal of Cy3-labeled probe MegPol436, specific for '*Candidatus* Megaira polyxenophila'; (b) signal of Fluorescein-labeled probe Rick_527, targeting members of the *Rickettsiaceae* family; (c) merged image, (a) + (b); (e) signal of Fluorescein-labeled probe HoloCar698, specific for *Holospora caryophila*; (f) merged image, (d) + (e). Bar: 10 µm.

**Figure 3:**
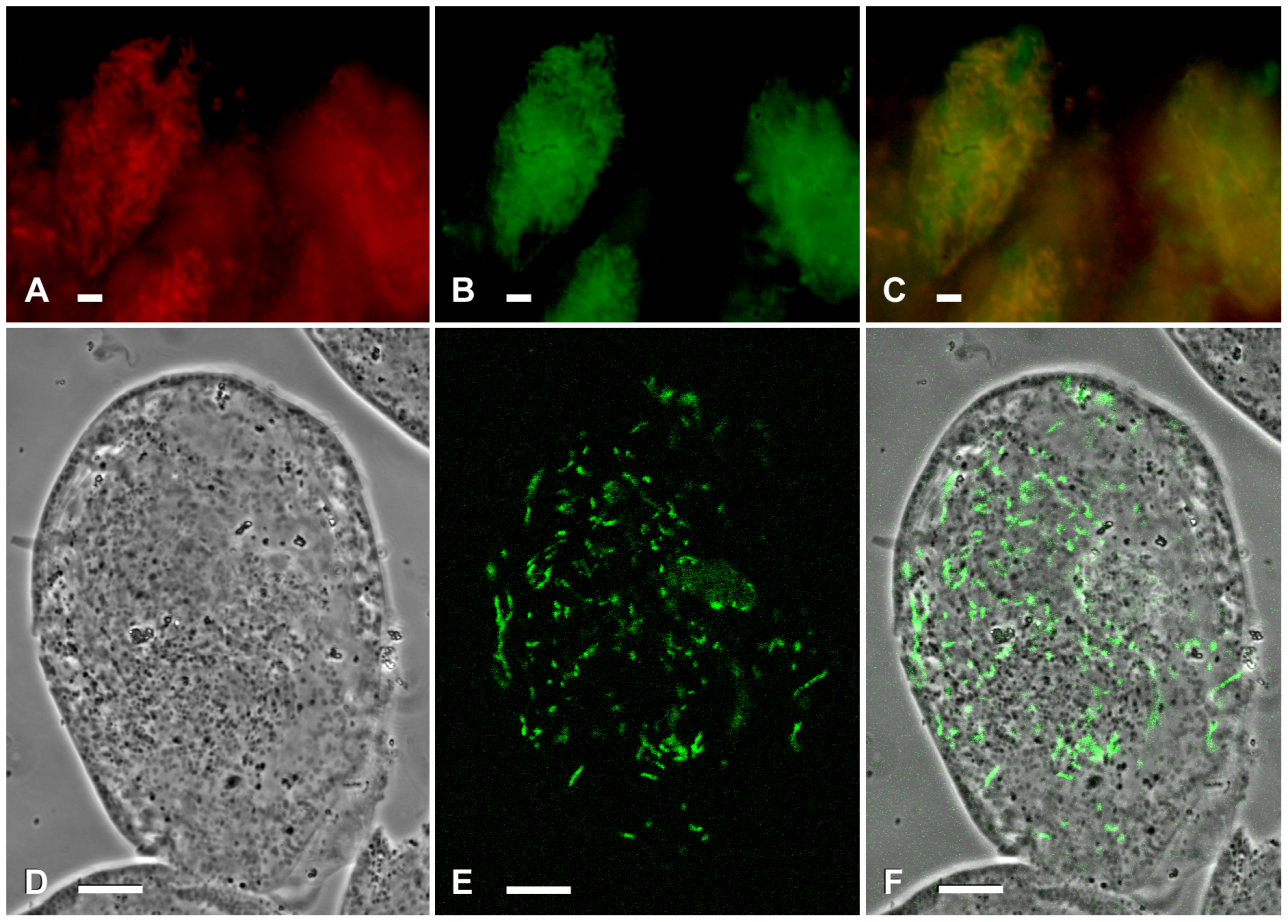
Fluorescence *in situ* hybridizations of *Spirostomum* sp. 72 and *Euplotes octocarinatus* FL(12)-VI. *Spirostomum* sp. 72 was fixed with 4% osmium tetroxide (a-b-c) and *Euplotes octocarinatus* FL(12)-VI was fixed with 4% formaldehyde in PBS (d-e-f). (a) signal of Cy3-labeled probe MegPol436, specific for '*Candidatus* Megaira polyxenophila' (macronuclei enhanced); (b) signal of Fluorescein-labeled probe EUB338 (macronuclei enhanced); (c) merged image, (a) + (b); (d) phase contrast; (e) signal of Fluorescein-labeled probe Rick_527, targeting members of the *Rickettsiaceae* family; (f) merged image, (d) + (e). Bar: 10 µm.

The endosymbionts were harboured within the host cytoplasm in case of *D. oligothrix* BOD9, DS12/4, and *E. octocarinatus* FL(12)-VI, and within the macronucleus in case of *P. caudatum* SH42 and *Spirostomum* sp. 72. When localized in the macronuclear compartment, bacteria have been often observed in tightly packed aggregations, while they displayed a homogeneous distribution when harboured in the cytoplasm (Figs. 2, 3).

All four newly described host strains were found to be infected by additional intracellular bacteria (Figs. 2, 3). *D. oligothrix* DS12/4 was associated with another yet-uncharacterized *Rickettsiaceae* species, as it showed additional positive signals with probe Rick_527 (used in double hybridization with probe MegPol436); *P. caudatum* SH42 was additionally infected by another macronuclear confined bacterium labelled by the probe designed for *Holospora caryophila*; *Spirostomum* sp. 72 hosted three intracellular bacteria: the macronucleus was occupied by the bacterium of interest together with *Caedibacter caryophilus* (positive signals with probe CC23a), and a third not characterized bacterium present in the cytoplasm (additional signal by probe EUB338). *E. octocarinatus* FL(12)-VI also harboured *Polynucleobacter*-like symbionts, identified by the genus-specific probe Poly_862, which have been recently described [35].

### Novel sister taxon to *Rickettsia*

A consensus sequence was generated for each group of clones and used for phylogenetic analyses and similarity matrix calculation. Phylogenetic reconstructions (Fig. 1) showed that the four novel sequences cluster within a pre-existing monophyletic clade. We named this clade *Candidatus* Megaira (corresponding to the Hydra group in [9], [10]). It is divided into following three subclades: *Candidatus* Megaira polyxenophila, *Candidatus* Megaira B, and *Candidatus* Megaira C (Fig 1). The *Candidatus* Megaira polyxenophyla subclade is composed of the four newly characterized sequences and the previously published sequences of the symbionts of *D. oligothrix* BOD9 [28], *Carteria cerasiformis*, and *Pleodorina japonica* [10]. It additionally contains seven sequences derived from the screenings of microbial communities: EF520417 from an acid-impacted lake [51], FJ612282 from lake Dongping [52], AF523878 from forested wetland [53], DQ223223 from subsurface water of the Kalahari Shield [54], JX105713 from bacterioplankton in an ornamental fish aquarium [55], JF828749 from waste water [56], and JN869203 from water of lake Taihu [57]. *Candidatus* Megaira subclade B comprises seven sequences, six sequences of eukaryotic-associated bacteria: FJ203077, associated with *Montastraea faveolata* [58], DQ395479 and DQ395439, associated with a deep-sea octacoral [59], HE648945-47 associated with *Bryopsis* sp. [23], and one sequence, HQ691997, derived from the screening of a microbial community of a stratified lagoon in the Clipperton atoll [60]. Finally, *Candidatus* Megaira subclade C comprises five sequences, three of which of eukaryotic associated organism: EF667896 and EF667899 associated with *Hydra oligactis* [20]; and GQ870455 associated with another ciliate, the fish parasite *Ichthyophthirius multifiliis* [15]. The remaining two sequences are again deriving from microbial community screenings, i.e. EF520410 from an acid-impacted lake [51], and CU466797 from the anoxic basin of a municipal wastewater treatment plant [61].

The *Candidatus* Megaira clade is robustly monophyletic, documented by good statistical values of bootstrap respectively Posterior Probabilities (Fig 1). According to topologies retrieved with all trees, this group branches as sister group of the genus *Rickettsia*. Basally located to these two groups are the other *Rickettsiaceae* genera, *Orientia* and '*Candidatus* Cryptoprodotis'. The tree has been rooted (according to [62], [63]) using the clade *Anaplasmataceae* plus *Midichloriceae* as outgroup. Also the subclades *Candidatus* Megaira polyxenophyla and Megaira B are robustly monophyletic. On the contrary, the monophyly of subclade *Candidatus* Megaira C is only moderately supported mainly because of the basally branching and rather divergent sequence CU466797.

Similarity percentages between sequences belonging to the *Candidatus* Megaira clade were calculated (Table 2). Almost all sequences belonging to this clade share a similarity higher than 95.0%. In the few cases in which the similarity drops down to 93.5% (e.g. sequence CU466797), the relevant sequence shows in any case a similarity higher than 95.0% with at least some members from a different *Candidatus* Megaira subclade. Within the *Candidatus* Megaira polyxenophyla subclade, similarity percentages are always above 98.4%; also in this case the more deviating sequences are derived from the screening of environmental libraries (sequences AF523878 and FJ612282). If similarity values are calculated only among the seven symbionts (present study, [10], [28]), they are always higher than 99.0%. The seven sequences forming the *Candidatus* Megaira subclade B reveal a similarity among themselves higher than 96.0%, for the five sequences forming the *Candidatus* Megaira subclade C it is higher than 95.1%, again sequence CU466797 being the more divergent one. Subclades B and C apparently comprise more heterogeneous sequences than subclade *Candidatus* Megaira polyxenophyla, although it contains more.

**Table 2:** Similarity matrix of bacterial SSU sequences.

	1	2	3	4	5	6	7	8	9	10	11	12	13	14	15	16	17	18	19	20	21	22	23	24	25	26
1. symb. of *D. oligothrix* BOD9, AJ630204	**100**																									
**2. symb. of ** ***P. caudatum*** ** SH42, FR822997**	**99.5**	**100**																								
3. symb. of *Carteria cerasiformis*, AB688628	**99.9**	**99.5**	**100**																							
4. derived from acid-impacted lake, EF520417	**99.3**	**99.0**	**99.3**	**100**																						
**5. symb. of ** ***Spirostomum*** ** sp. 72, FR822998-FR823000**	**100**	**99.6**	**100**	**99.4**	**100**																					
**6. symb. of ** ***E. octocarinatus*** ** FL(12)-VI, FR823004-FR823007**	**99.9**	**99.5**	**99.9**	**99.3**	**100**	**100**																				
7. derived from water of lake Dongping, FJ612282	**98.4**	**98.1**	**98.4**	**97.9**	**98.5**	**98.4**	**100**																			
8. symb. of *Pleodorina japonica*, AB688629	**99.4**	**99.0**	**99.4**	**98.9**	**99.5**	**99.4**	**98.0**	**100**																		
9. derived from a fish aquarium, JX105713	**99.4**	**99.0**	**99.4**	**98.9**	**99.5**	**99.4**	**98.0**	**99.7**	**100**																	
10. derived from waste water, JF828749	**99.4**	**99.0**	**99.4**	**98.9**	**99.5**	**99.4**	**98.0**	**99.6**	**99.6**	**100**																
**11. symb. of ** ***D. oligothrix*** ** DS12/4, FR823001-FR823003**	**99.4**	**99.0**	**99.4**	**98.9**	**99.5**	**99.4**	**98.0**	**99.4**	**99.4**	**99.4**	**100**															
12. derived from forested wetland, AF523878	**99.0**	**98.6**	**99.0**	**98.5**	**99.1**	**99.0**	**98.6**	**98.9**	**98.9**	**98.9**	**99.0**	**100**														
13. derived from water of lake Taihu, JN869203	**99.4**	**99.0**	**99.4**	**98.9**	**99.5**	**99.4**	**98.0**	**99.4**	**99.4**	**99.4**	**99.6**	**99.0**	**100**													
14. derived from subsurface water of Kalahari, DQ223223	**99.5**	**99.1**	**99.5**	**99.0**	**99.6**	**99.5**	**98.1**	**99.3**	**99.3**	**99.3**	**99.5**	**98.9**	**99.5**	**100**												
15. symb. of *Bryopsis* sp., HE648945	94.7	94.4	94.7	94.2	94.9	94.7	94.1	94.9	94.9	94.7	94.8	94.3	94.8	94.9	**100**											
16. symb. of *Bryopsis* sp., HE648947	94.8	94.4	94.8	94.3	95.0	94.8	94.2	95.0	95.0	94.8	94.9	94.4	94.9	95.0	**99.8**	**100**										
17. symb. of *Montastraea faveolata*, FJ203077	94.2	93.9	94.2	93.7	94.4	94.2	93.6	94.2	94.2	94.1	94.2	93.6	94.1	94.4	**98.1**	**98.2**	**100**									
18. symb. of *Bryopsis* sp., HE648946	95.9	95.6	95.9	95.4	96.1	95.9	95.0	95.9	95.9	95.9	96.0	95.5	96.0	96.1	**97.6**	**97.6**	**97.0**	**100**								
19. symb. of deep-sea octacoral, DQ395479	94.7	94.3	94.7	94.2	94.8	94.7	93.7	94.5	94.5	94.5	94.7	94.2	94.7	94.8	**96.8**	**96.9**	**96.5**	**97.3**	**100**							
20. symb. of deep-sea octacoral, DQ395439	94.4	94.0	94.4	93.9	94.5	94.4	93.4	94.2	94.2	94.2	94.4	93.8	94.4	94.5	**96.5**	**96.6**	**96.2**	**97.0**	**99.4**	**100**						
21. derived from a stratified lagoon, HQ691997	94.8	94.4	94.8	94.4	94.9	94.8	93.9	94.7	94.7	94.7	94.7	94.4	94.8	95.0	**96.1**	**96.1**	**95.5**	**96.7**	**96.3**	**96.0**	**100**					
22. symb. of *Hydra oligactis*, EF667899	95.4	95.0	95.4	94.9	95.5	95.4	94.4	95.2	95.2	95.2	95.4	95.0	95.4	95.6	95.5	95.5	95.0	96.7	95.0	94.7	95.2	**100**				
23. symb. of *Hydra oligactis,* EF667896	95.6	95.2	95.6	95.1	95.7	95.6	94.7	95.5	95.5	95.5	95.6	95.3	95.6	95.8	95.7	95.8	95.2	96.9	95.2	95.0	95.5	**99.6**	**100**			
24. derived from acid-impacted lake, EF520410	95.9	95.5	95.9	95.4	96.0	95.9	95.0	95.8	95.8	95.8	95.9	95.5	95.9	96.1	96.0	96.1	95.5	97.2	95.5	95.3	95.8	**99.3**	**99.6**	**100**		
25. symb. of *Ichthyophthirius multifiliis,* GQ870455	95.6	95.3	95.6	95.2	95.7	95.6	94.8	95.5	95.5	95.5	95.5	95.2	95.5	95.5	94.7	94.8	94.4	95.3	95.3	95.0	95.4	**96.6**	**96.8**	**97.1**	**100**	
26. derived from wastewater treatment plant, CU466797	94.0	93.6	94.1	93.5	94.1	94.1	93.6	94.1	94.1	94.0	94.1	93.7	94.1	94.0	94.9	95.0	94.4	94.9	94.8	94.5	94.8	**95.1**	**95.4**	**95.5**	**95.5**	**100**

Numbers represent similarity percentages. Presented sequences were either obtained from bacterial symbionts (symb.) or are environmental sequences deriving from screenings of microbial communities of diverse humid or aquatic habitats. Highlighted in bold are the sequences obtained in this study. Numbers reported in bold are similarity values which indicate that the respective sequences (1–14; 15–21; 22–26) belong to the same clade.

### Ultrastructural observation of symbionts

**Figure 4:**
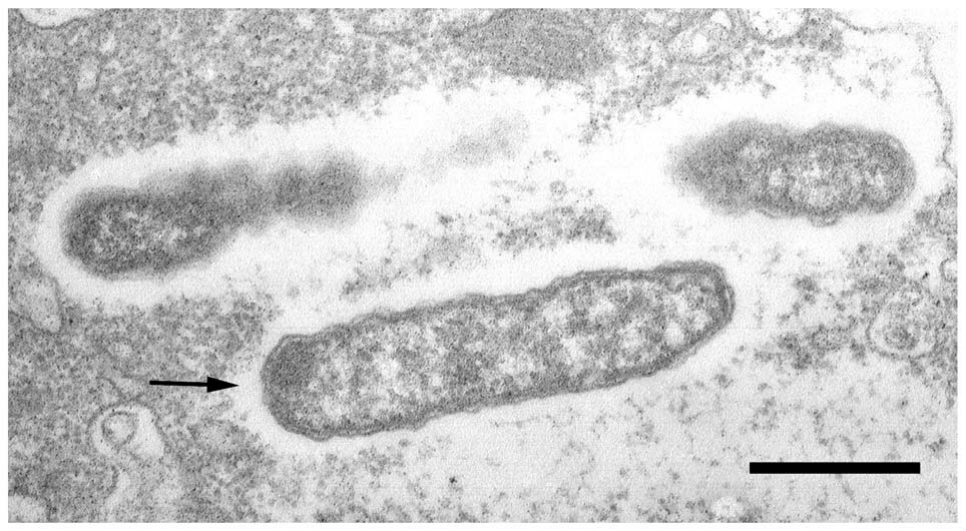
Transmission electron microscopy of *Diophrys oligothrix* BOD9 harbouring '*Candidatus* Megaira polyxenophila'. One whole bacterium and two partial ones are visible, all referable to morphotype I. Bacteria are free in the cytoplasm of the host. The arrow indicates the clear zone (halo) surrounding the cells. Bar: 1 µm.

In hosts infected by more than one endosymbiont, it is generally difficult to unambiguously discriminate those bacteria on ultrastructural level. Therefore the ultrastructural description of the investigated bacteria relies exclusively on those present in the cytoplasm of *D. oligothrix* BOD9, which harbours no additional symbionts. Bacteria generally appeared as rod-shaped, measuring 1.5–1.6 µm in length and 0.3–0.4 µm in width (Fig. 4). Elongated forms (likely dividing cells), attaining up to 3.2 µm, were occasionally observed (data not shown). The endosymbionts presented a typical Gram-negative organization (outer membrane – periplasmic space – inner membrane) and no visible pili or flagella. The cytoplasm was substantially homogeneous, though the regions close to the membrane appeared slightly more electrondense than the inner part (Fig. 4). Nucleoids or other intracellular structures were never observed. Some bacteria showed a deviating morphology, with a more electrondense cytoplasm and a reduced size (data not shown). The latter can be referred to as “morphotype II”, while the more common type as “morphotype I” [28]. Different morphotypes similar to those described here have been also described in *Rickettsia prowazekii* and *Rickettsia rickettsii* (reviewed by [64]). All morphotypes were observed as occurring freely in the cytoplasm; they appeared to be surrounded by a lucent zone (halo) not delimited by any membranes (Fig. 4). An electron translucent area surrounding the bacteria in their host cells has been observed also for other symbiotic *Rickettsiales* [18], [65]. The overall appearance of the bacteria was referable to the description reported by Vannini et al. [28].

## Discussion

The here described bacteria dwell not only as endosymbionts in different hosts and in different cell compartments but, as discovered so far, most often in co-occurrence with other bacterial symbionts. This finding indicates a great flexibility in adaptation to particular host's intracellular environments. At present, no information is available if these co-occurring endosymbionts interact or compete for their host's resources. It is very probable that the kind of interaction strongly depends on the involved bacteria. In this context it should be mentioned that the additionally found bacteria seem to cover the complete range of symbiotic interactions: from parasitic like *Caedibacter caryophilus* [66] in *P. caudatum* SH42 to mutualistic *i.e. Polynucleobacter*-like [35] in *E. octocarinatus* FL(12)-VI. Further studies, first of all a precise identification of the additional symbionts, will help in better understanding some of these intriguing matters. However, the probably transient nature of the infections leads us to hypothesize a possible opportunistic or parasitic lifestyle of the newly described bacteria.

Similarity percentages of the SSU rRNA gene sequences belonging to the subclade *Candidatus* Megaira polyxenophyla are very close to the nowadays assumed threshold (98.7%) to define separated bacterial species in the absence of DNA-DNA hybridization data [67]. Accordingly, molecular data support the affiliation of the here described rickettsial endosymbionts and those previously characterized [10], [28] to a single bacterial species, which is proposed as ‘*Candidatus* Megaira polyxenophila’. It is specifically recognized by probe MegPol436 that matches *in silico* all members of the subclade and obtained satisfying *in situ* hybridisation results with the organisms characterized in the present study.

Similarity values higher than 95.0% are shared between almost all sequences comprised within the *Candidatus* Megaira clade, supporting the hypothesis that the whole clade is represented by a candidate new genus for which the name ‘*Candidatus* Megaira’ is proposed. Sequences encompassed in *Candidatus* Megaira subclade B and C (Fig. 1) show a higher diversity among themselves in respect to ‘*Candidatus* Megaira polyxenophila’, thus suggesting the presence of more than one species within both subclades.

Phylogenetic reconstruction (Fig. 1) reveals a broad and complex host range for '*Candidatus* Megaira polyxenophila' that has been found as endosymbiont of seven hosts belonging to two eukaryotic supergroups: SAR and Archaeplastida [68]. In total, the candidate genus 'Megaira' is presently found in association with 16 different hosts belonging to three eukaryotic supergroups including also Opisthokonta. The actual number of potential natural hosts is probably even higher if we consider the fact that *Rickettsiales* cannot exist as stable free-living forms and probably also '*Candidatus* Megaira' species share this feature. Sequences from screenings of microbial communities encompassed in the *Candidatus* Megaira clade are likely derived from unrecognized eukaryotic hosts.

The fact that all the presently recognized hosts of '*Candidatus* Megaira polyxenophila', except the two *D. oligothrix* strains (DS12/4 and BOD9), are phylogenetically far related seems to suggest that this symbiont is not inherited from a common ancestor; on the contrary it supports the hypothesis that the bacteria colonized each host separately. Other authors concluded from recent phylogenetic reconstructions (e.g. [10]) that the presence of closely related *Rickettsiaceae* endosymbionts in evolutionary distant hosts can be most easily explained accounting for horizontal transmission [10], [69], [70]. Moreover, the high sequence identity values between different strains of '*Candidatus* Megaira polyxenophila', retrieved in different hosts, indicate that these specific host shifts took place in relatively recent times. These results allow developing a new hypothesis contrary to the supposed mechanisms of diversification of rickettsial organisms.

Associations with *Rickettsiaceae* bacteria have been reported so far for more than fifty different eukaryotic species, mainly represented by terrestrial arthropods [9]. The most parsimonious interpretation about their radiation has therefore been an “arthropod first” view, in which the common ancestor of *Rickettsiaceae* already infected terrestrial arthropods and occasionally switched to other kinds of eukaryotes during its evolutionary history [9]. This view is regarded as the most common scenario according to a recent review on rickettsial evolution, nevertheless presenting an alternative: the first host cell of *Rickettsia* might have been an amoeba [71]. Although our results and the numerous recent reports of *Rickettsiaceae* bearing protists [12]–[19] do neither proof nor contradict these hypotheses, they seem to indicate a major role of protists as ancestral host organisms.

Regardless of the ancestral state, the actual transmission mechanism of the basal *Rickettsiaceae* remains unclear. Obviously, the route of *Rickettsia* species inhabiting hematophagous arthropods to infect vertebrate hosts is not available to bacteria inhabiting protists, although the infection of plants respectively plant phloem seems to follow a comparable mechanism [22], [70]. Noticeable, in both cases the bacterial transfer is a result of the feeding behaviour of the host. Likewise, a possible scenario for horizontal transmission of basal *Rickettsiaceae* involves food web interactions, but specifically within aquatic environments as catalysts for host shifting events. Those habitats could facilitate horizontal transmission of rickettsial infections between hosts which share similar ecological niches, as it possibly happened in case of '*Candidatus* Megaira polyxenophila'. Two routes of horizontal transmission could be hypothesized. The first would involve the release of bacteria capable to actively invade new hosts; in support of this possibility are the cases of two autotrophic algal species harbouring '*Candidatus* Megaira polyxenophila' [10]. A second transmission pathway would involve the transfer of '*Candidatus* Megaira polyxenophila' among trophic levels. The filter feeding behaviour of ciliates and their documented capability to uptake symbiotic/parasitic bacteria via this system [72] provides an alternative route. Accordingly, a basally ranked organism within the aquatic food web (e.g. a protist) may act as natural reservoir and vector for *Rickettsiaceae* bacteria, the infection being subsequently transmitted up to higher trophic levels by swallowing of infected preys, as it happens in other *Rickettsiales* (e.g. *Neorickettsia* spp. [73]). This process could take place most easily in aquatic environments, where grazing- or filter-feeding strategies (allowing the swallowing of protists) are exploited by a variety of invertebrates. On the contrary, the eventual uptake of protists by terrestrial arthropods would be nearly an accidental phenomenon. Therefore, it could be speculated that aquatic protists might have contributed to the spread of rickettsial bacteria towards other eukaryotic lineages, including arthropods. The fact that some terrestrial rickettsiae-harbouring arthropods contemplate one or more aquatic stages during their life history (e.g. *Lutzomya* spp., *Limonia* spp.) may account for explaining the passages between water-bounded hosts to terrestrial ones, and a subsequent radiation among terrestrial arthropods. This hypothesis would suggest a major role of protists in the spreading processes of *Rickettsiaceae* as well as in their evolutionary history.

### Description of '*Candidatus* Megaira polyxenophila' gen. nov. sp. nov

Megaira polyxenophila (Me.ga'i.ra. G. N. fem. “the Envious”, one of the Erynes in the Greek mythology [also intended as an ironic compliment to one of the authors], po.ly.xe.no'.phi.la, G. adj. polys, many, G. N. xenòs, host, G. v. phìlein, to love, which loves many hosts).

Rod-shaped intracellular bacteria of variable size, generally measuring 1.45 µm in length (elongated forms attaining up to 3.2 µm) and 0.3 µm in width, with a typical Gram-negative structure. Cytoplasm clear, more electrondense near the periphery (morphotype I) or greatly electrondense and homogeneous (morphotype II). Absence of either intra- or extracellular structures. Bacteria were never observed surrounded by additional membranes. Often surrounded by an electron lucent zone (halo). Occurs in Ciliophora, localized in the macronucleus (*Paramecium*, *Spirostomum*) or the cytoplasmic compartment (*Diophrys*, *Euplotes*) and in the cytoplasm of Archaeplastida (*Carteria*, *Pleodorina*). Likely able to colonize other eukaryotic hosts. Phylogenetic position of this organism within the *Alphaproteobacteria*, order *Rickettsiales*, family *Rickettsiaceae*, representing the type species of the candidate genus 'Megaira'. Type strain: symbionts of the ciliate *Diophrys oligothrix* BOD9, in which the novel species was primarily discovered and characterized. The basis of assignment to a novel genus within *Rickettsiaceae* was the sequence of the SSU rRNA gene of the type strain (European Nucleotide Archive accession number AJ630204) and recognition with the specific oligonucleotide probe MegPol436 (*5* ′-TTATCTTTCCAACTAAAAG-*3*′). The free-living ciliated Protist *Diophrys oligothrix* BOD9 (Ciliophora, Spirotrichea), host of type strain, was isolated from a brackish environment in Boderne (Bornholm, Baltic Sea).
